# Supplementary figures and images for: Murine modeling of menstruation identifies immune correlates of protection during Chlamydia muridarum challenge
Source: PLoS Pathog. 2025 Jun 6;21(6):e1012276. doi: 10.1371/journal.ppat.1012276 (PMC12176300; doi:10.1371/journal.ppat.1012276)

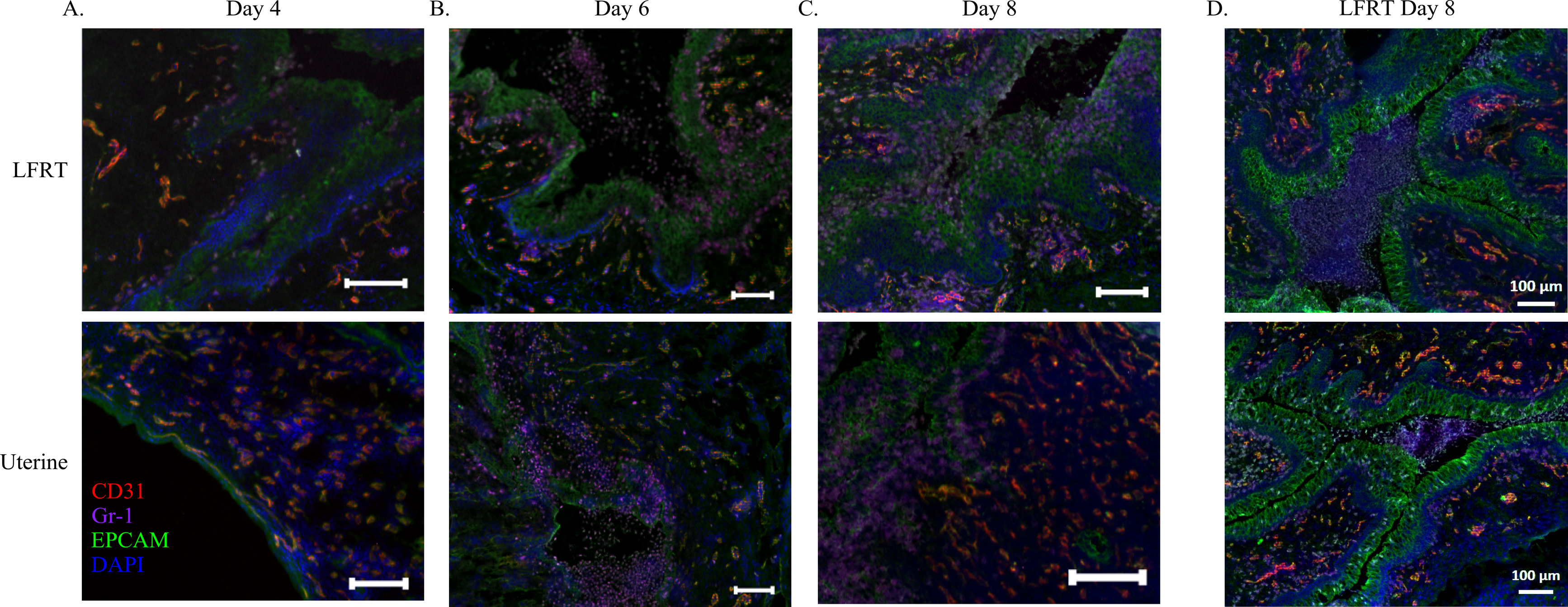

Supplement: S1 Fig — Fluorescence microscopy images taken at 20x magnification identify blood vessels (CD31 expression in red), myeloid cells (Gr-1 expression in purple), epithelial cells (EPCAM expression in green), and DAPI staining (cell nuclei blue) in the lower FRT (LFRT top panels), and uterine horns (bottom panels) at day 4, (B). day 6, and (C). day 8 of pseudopregnancy. (D). Additional images of the LFRT on day 8. (A-C). The white scale bars indicate 100 μm length. (TIF) [file ppat.1012276.s001.tif]

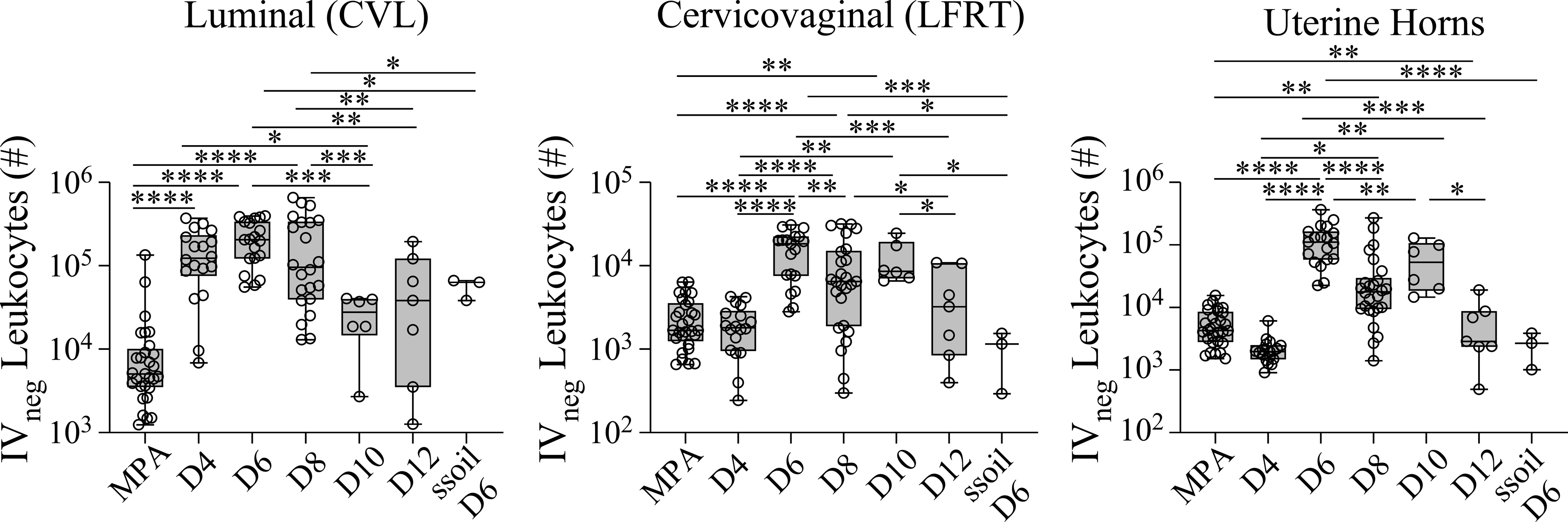

Supplement: S2 Fig — Models used to compare a difference of means were fit using multiple comparisons with FDR testing: *p ≤ 0.05, **p < 0.01, ***p < 0.001, ****p < 0.0001. (TIF) [file ppat.1012276.s002.tif]

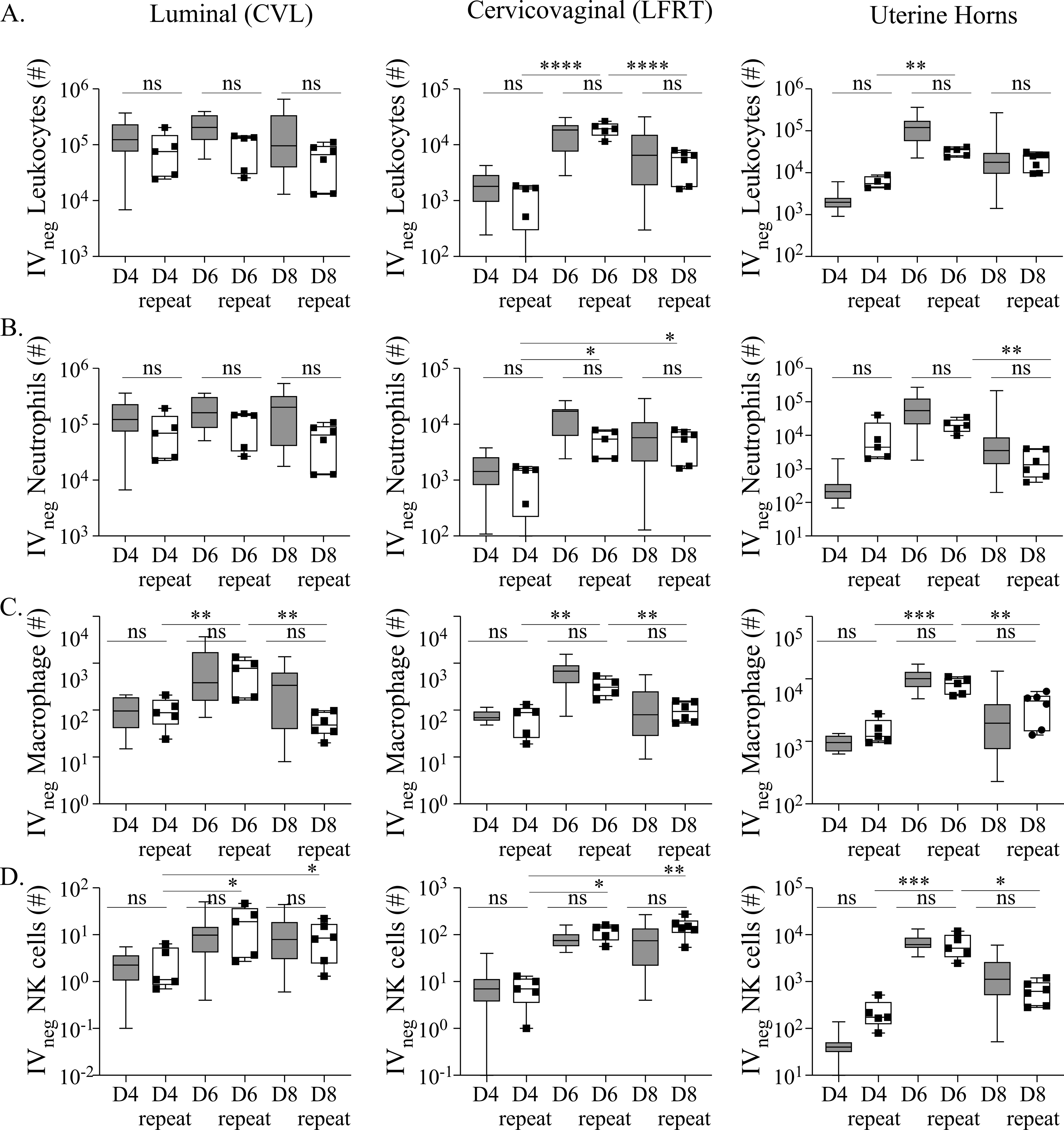

Supplement: S3 Fig — Neutrophils, (C). Macrophage, and (D). NK cells from indicated FRT tissues are plotted as box and whiskers graphs and compared by the first (taken from Figs 2 and S2) or second (white with black squares representing individual values labeled as a repeat) pseudopregnancy cycle. Models used to compare a difference of means were fit using multiple comparisons with FDR testing: *p ≤ 0.05, **p < 0.01, ***p < 0.001, ****p < 0.0001. ns-not significant. (TIF) [file ppat.1012276.s003.tif]

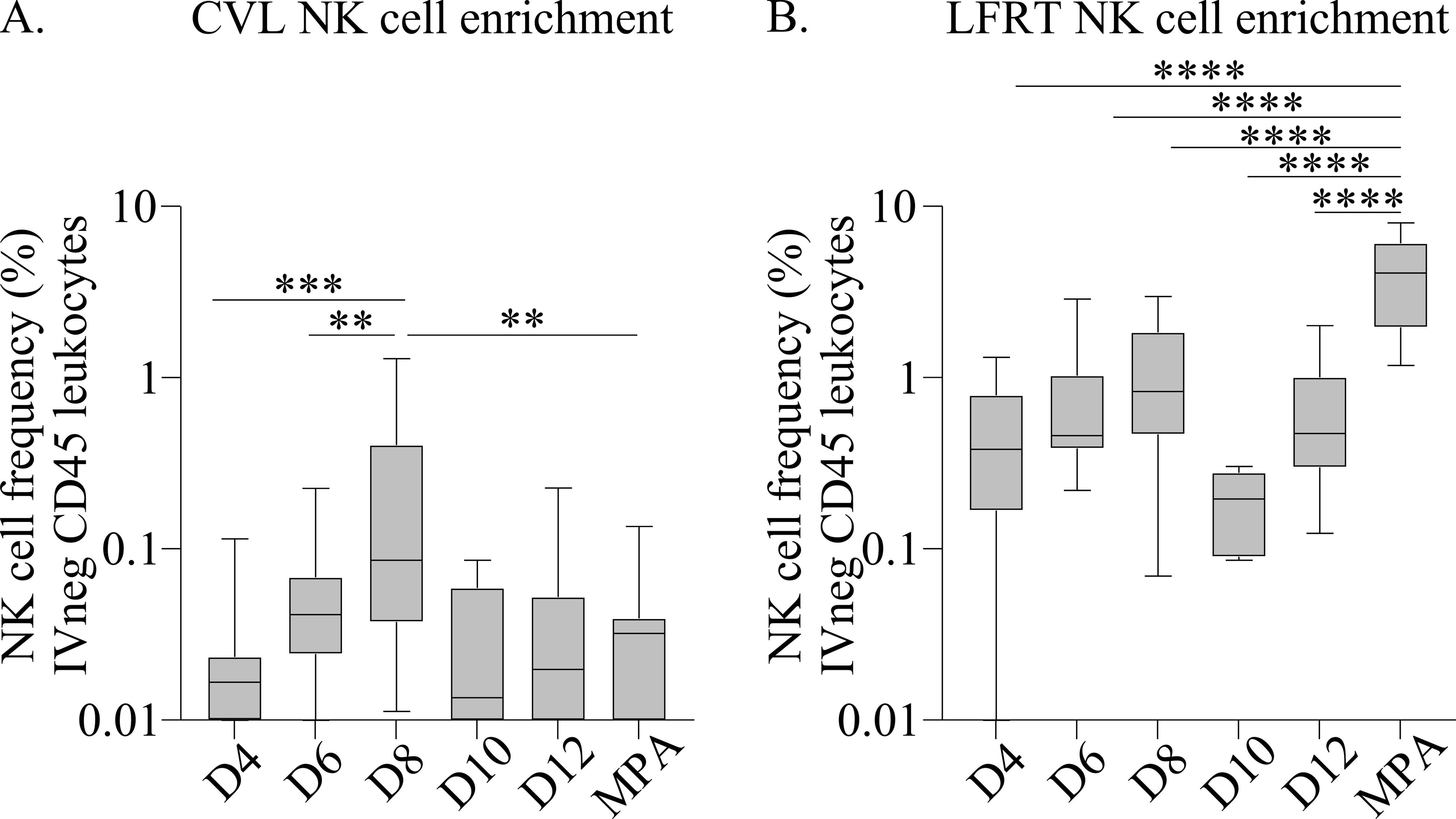

Supplement: S4 Fig — CVL or (B). LFRT at indicated days of pseudopregnancy or in MPA-treated mice. Models used to compare a difference of means were fit using multiple comparisons with FDR testing: **p < 0.01, ***p < 0.001, ****p < 0.0001. (TIF) [file ppat.1012276.s004.tif]

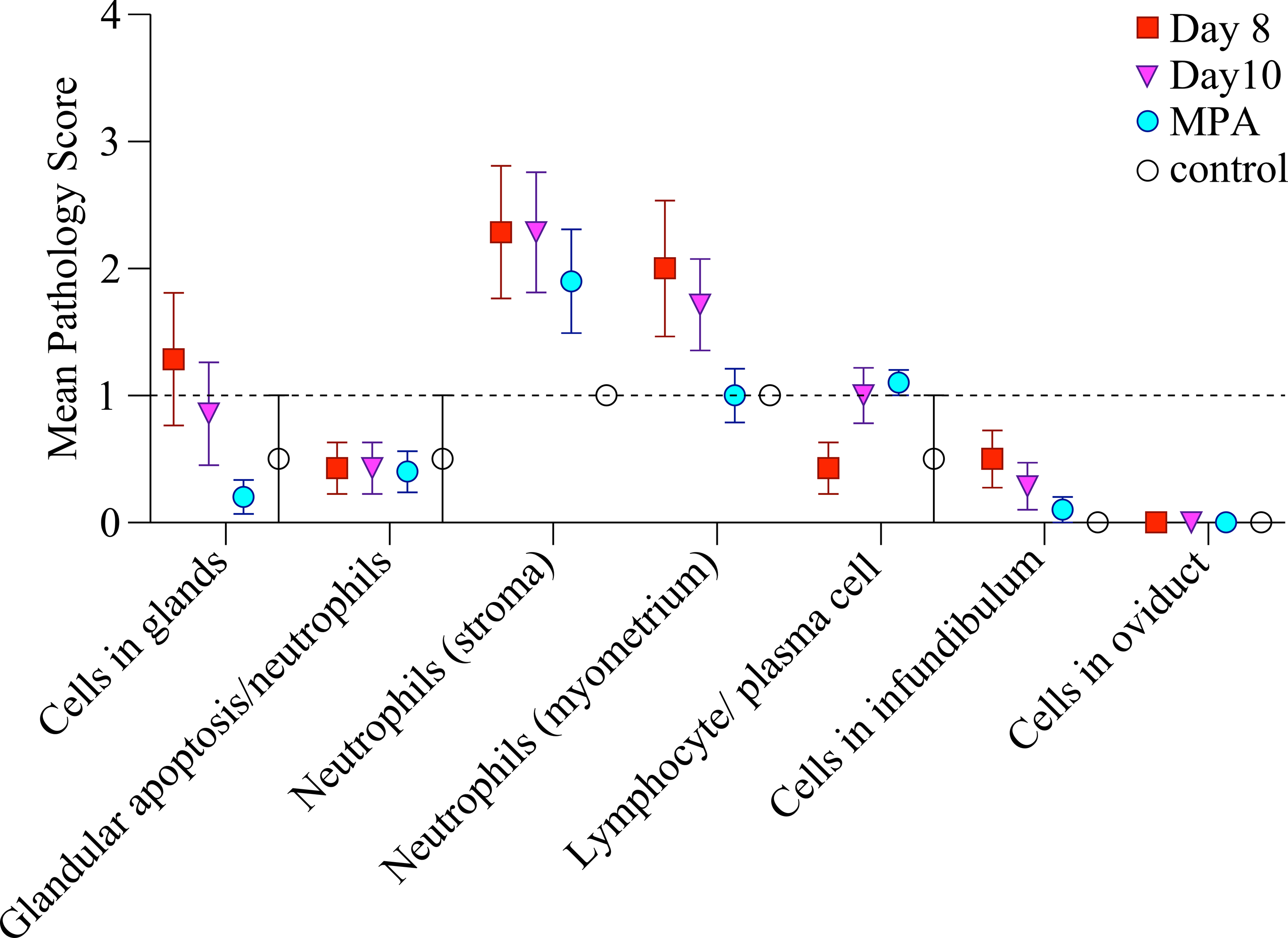

Supplement: S5 Fig — Groups: day 8 of pseudopregnancy (n = 7), day 10 of pseudopregnancy (n = 7), MPA (n = 10), and control mice (naïve n = 2). Tissues scoring 0–1 identify little to no detection, 2–3 identify moderate detection, and 4 identify severe infiltration or damage. No fibrosis or tissue damage, including oviduct dilation, was detected. Models used to evaluate score deviation (against a value of 1) were fit using Wilcoxon rank sum tests. No group scores were significantly increased. (TIF) [file ppat.1012276.s005.tif]

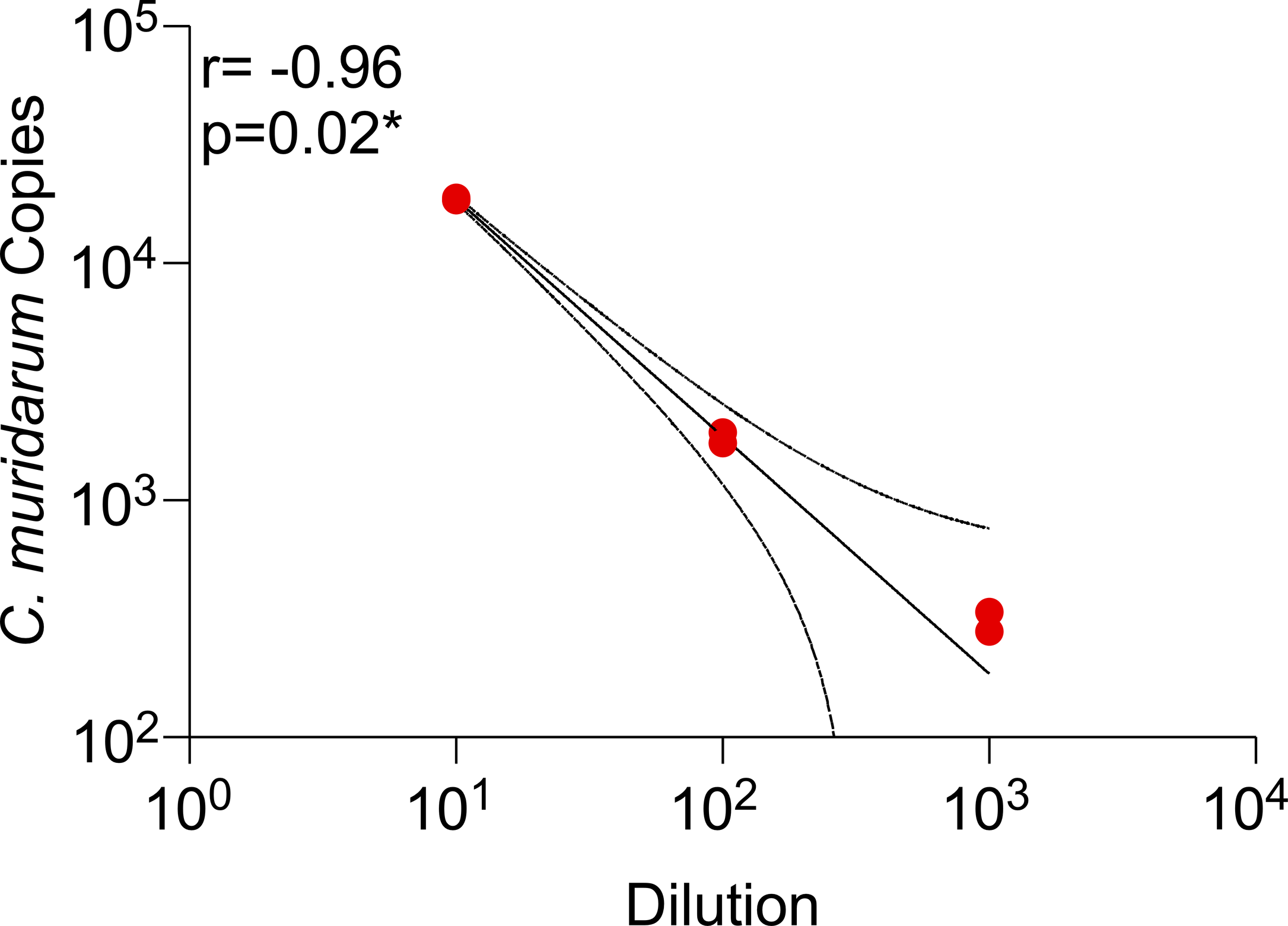

Supplement: S6 Fig — Distributions were tested by Spearman’s correlations. (TIF) [file ppat.1012276.s006.tif]
