# Supplementary material for: Murine modeling of menstruation identifies immune correlates of protection during Chlamydia muridarum challenge
Source: PLoS Pathog. 2025 Jun 6;21(6):e1012276. doi: 10.1371/journal.ppat.1012276 (PMC12176300; doi:10.1371/journal.ppat.1012276)
Supplement: S1 Table — (DOCX) [file ppat.1012276.s007.docx]

|  | MPA (pg/mL) | Day 4 (pg/mL) | Day 6 (pg/mL) | Day 8 (pg/mL) | Day 10 (pg/mL) | Day 12 (pg/mL) |
| --- | --- | --- | --- | --- | --- | --- |
| IL15 | 18.825 (3.489) | 96.336 (36.655) | 218.936 (22.966) | 90.755 (24.473) | 181.386 (47.04) | 32.26 (14.009) |
| IL-17A | 1.242 (0.185) | 9.915 (2.939) | 80.811 (12.351) | 9.481 (2.765) | 10.151 (3.025) | 2.633 (0.822) |
| IL-27P28 | 1.658 (0.421) | 4.571 (1.316) | 26.045 (4.042) | 12.289 (5.05) | 16.329 (5.771) | 2.016 (0.623) |
| IL-33 | 13.544 (4.126) | 7.508 (3.059) | 5.522 (0.594) | 11.091 (3.077) | 9.911 (3.03) | 1.881 (0.717) |
| IL-9 | 0.873 (0.572) | 1.856 (.0878) | 4.096 (1.016) | 1.587 (0.863) | 3.234 (2.156) | 1 (0.521) |
| IP-10 | 8.959 (1.146) | 15.375 (4.145) | 845.045 (216.52) | 103.941 (33.999) | 119.646 (49.228) | 5.081 (1.767) |
| MCP-1 | 2.056 (0.4) | 8.631 (4.181) | 17.370 (2.771) | 19.829 (5.712) | 68.625 (42.208) | 53.521 (39.849) |
| MIP-1α | 12.803 (1.441) | 169.677 (65.429) | 329.283 (54.306) | 147.607 (67.654) | 253.184 (103.611) | 24.035 (13.966) |
| MIP-2 | 132.679 (21.303) | 1188.208 (244.673) | 1936 (24.36) | 997.099 (250.718) | 1461.614 (250.464) | 530.557 (202.535) |
| IFNγ | 0.049 (0.012) | 0.2 (0.134) | 104.177 (20.15) | 1.691 (0.599) | 16.806 (13.003) | 0.201 (0.114) |
| IL-10 | 0.733 (0.223) | 16.69 (8.08) | 64.891 (14.663) | 22.646 (9.159) | 30.386 (14.889) | 0.882 (0.258) |
| IL12p70 | 1.339 (0.494) | 3.596 (1.177) | 30.338 (4.65) | 7.966 (3.07) | 13.117 (4.122) | 1.753 (0.788) |
| IL-1β | 393.715 (55.892) | 285.274 (75.729) | 1341.195 (174.339) | 1163.974 (695.134) | 1665.124 (1178.348) | 157.367 (63.492) |
| IL-2 | 0.038 (0.012) | 0.071 (0.017) | 0.766 (0.13) | 0.599 (0.219) | 0.57 (0.253) | 0.063 (0.03) |
| IL-4 | 0.169 (0.033) | 0.089 (0.025) | 0.495 (0.092) | 0.39 (0.131) | 0.249 (0.048) | 0.216 (0.076) |
| IL-5 | 0.389 (0.056) | 0.615 (0.219) | 1.867 (0.492) | 1.356 (0.527) | 1.071 (0.373) | 0.14 (0.035) |
| IL-6 | 2.16 (0.388) | 42.375 (14.848) | 7741.421 (1358.467) | 714.485 (250.752) | 2950.566 (1589.784) | 9.655 (5.112) |
| CXCL1 | 62.722 (14.326) | 137.711 (86.099) | 846.746 (138.84) | 519.134 (188.805) | 882.652 (267.625) | 37.582 (12.072) |
| TNFα | 8.714 (0.987) | 295.473 (82.585) | 2882.011 (357.084) | 367.174 (148.467) | 371.82 (127.41) | 49.419 (20.185) |
