# Supplementary material for: Murine modeling of menstruation identifies immune correlates of protection during Chlamydia muridarum challenge
Source: PLoS Pathog. 2025 Jun 6;21(6):e1012276. doi: 10.1371/journal.ppat.1012276 (PMC12176300; doi:10.1371/journal.ppat.1012276)
Supplement: Attachment — Submitted filename: PLOS_RESPONSE_FINAL.docx [file ppat.1012276.s009.docx]

**Response to Reviewers**

**Reviewer #1**:

The authors created the pseudopregnancy condition in naïve C57Bl/6 mice (Fig. 1) and monitored inflammatory cell and cytokine distributions (Fig. 2) in the lumen and different genital tissues along the pseudo-menstruation process using the MPA-treated mice as the control. They found that mice were resistant to C. muridarum infection until day 10 after pseudo-menstruation induction (Fig. 4). The increased susceptibility to C. muridarum infection on day 10 correlated with significantly decreased levels of 7 cytokines (IP-10, IL-5, IL-1b, CXCL1, IFNg, IL-6& IL-27p28) and leukocytes including neutrophils, macrophages, and NK cells (Fig. 5, no data on other cell types). Depleting with an anti-NK1.1 antibody significantly reduced NK cells and increased mouse susceptibility to C. muridarum infection on day 8 after induction of pseudo-menstruation (Fig. 6). Thus, the authors claimed that “murine modeling of menstruation identifies immune correlates of protection during C. muridarum challenge”. The main issue with this manuscript is that the same findings have been repeatedly published using MPA-treated mice, which is a much easier model to work with. In addition, some data conflicted with each other, and not all of the data presented in the manuscript supported the authors’ claims.

*We thank the Reviewer for communicating that our main objectives in the original manuscript draft were unclear, and this might lead to confusion regarding our results and interpretations. In response to the Reviewer’s concern, we have performed major writing revisions to the manuscript and added additional experimental data. We have clarified throughout the text that this study was focused on identifying immune correlates of protection against chlamydia infection in the FRT specifically under menstrual cycle regulation. We believe these revisions have strengthened the novelty and impact of our findings.*

*We agree with the Reviewer that our immune profiling of pseudopregnancy was focused on the predominant innate immune cell populations in the FRT and this approach led to numerous interesting discoveries, many of which we intend to pursue in future studies. We focused specifically on the day 8 time point of pseudopregnancy in this manuscript due to our discovery of early and sustained protection only following day 8 challenge, as shown in revised Figure 4. In the pseudopregnancy model, we found a strong correlation between NK cell populations that are enriched at the barrier site of pathogen exposure and protection from C. muridarum challenge. NK cells, as the Reviewer notes, have been shown to be important in the early immune response to C. muridarum in MPA-treated mice, and this background knowledge provided important rationale that helped to inform our hypothesis that the menstrual cycle regulates periodic NK cell recruitment into the site of sexual exposure where these cells can provide rapid immune protection.*

*Regarding the Reviewer’s concern that our conclusions of FRT NK cells as an immune correlate under menstrual cycle regulation are too generalized, we have added a transcriptomic comparison of FRT NK cells, directly comparing the day 8 time point with MPA-treated mice in revised Fig. 6. These data show that FRT NK cells are distinct populations under these conditions. Specifically, day 8 FRT NK cells were enriched for programming associated with effector responses, while the FRT NK cells in MPA-treated mice are consistent with NK cells during homeostatic conditions. Additionally, to better understand why the day 8 time point was protective, we have added supplemental data (SFig 4) showing that luminal NK cells are uniquely enriched from the leukocyte pool only at day 8.*

*We believe these new data provide mechanistic insights that explain the enhanced protection we observe at day 8 of pseudopregnancy, strengthen our overall conclusions regarding the impact of the menstrual cycle on infection risk, and support future investigations into these unique FRT NK cell populations and their contributions to immunity during the menstrual cycle.*

1. Figure 1 describes the pseudopregnancy model of menstruation. No new finding was presented. Panels F-H seemed to show the highest level of white blood cells circulating to the uterine horn tissues on day 8 after pseudopregnancy induction. However, it is not clear whether this was due to more circulation by the intravenously injected anti-CD45 antibody or the hematogenously labeled CD45+ cells. It is necessary to identify the subtypes of the FITC+ and/or PE+ cells if the authors want to claim that NK cells are among the recruited cells.

*We thank the Reviewer for their comments on strengthening Figure 1. In response, we have added new data in the revised Figure 1 I&J, Figure 3 E&F, and new Supplemental Figure 3, demonstrating the ability to induce consecutive cycles with the pseudopregnancy model. We believe this exciting new finding supports our overall conclusions in this manuscript, as the immune changes we detect are consistent. Moreover, we believe these additional data further justify the inclusion of this model in future studies aimed at understanding sex hormone dynamics and immune baseline states (demonstrated in revised Figure 3E&F).*

*In regard to the Reviewer’s comment on our use of intravital vascular (IV) labeling, we have revised Figures 1 and 2 and our description of the results to better clarify our approach. IV labeling allows us to distinguish circulating immune cells from tissue-localized immune cells (including recently recruited tissue localized cells); please see PMID: 24385150. The measurement of IV-labeled (i.e. circulating) leukocytes shown in Figure 1H is determined by staining with both the IV-labeled antibody in vivo and ex vivo staining with the same pan-leukocyte antibody conjugated to a different fluorochrome). As demonstrated in revised Figure 1G, we can identify increased vascularization of the uterine horns on day 8 due to the substantial increase in circulating leukocytes detected. In response to the Reviewer’s comment, we have revised Figure 1 and the description in the results to make this information more clear to the reader. Furthermore, to provide additional support for our findings in Figure 1H, we have added immunofluorescence microscopy in revised Figure 2A-D to visually distinguish tissue-localized immune cells and vasculature in the FRT over pseudopregnancy.*

*To identify the subtypes of immune cells recruited during the menstrual cycle, we apply this same IV labeling approach to distinguish tissue-localized leukocyte populations from those circulating in the vasculature. Although it is possible that some IV-labeled leukocytes may have extravasated into the tissues during endometrial remodeling (as might occur during severe acute infections) between the time frame of intravenous anti-CD45 PE-CF594 injection and euthanasia (at least 15 minutes as described in the Materials and Methods), this number would be nominal (PMID:24385150) and thus unlikely to alter our findings at day 6 and day 8 of pseudopregnancy.*

2. Figure 2, panel B, what does “iVneg” displayed along the Y-axis mean? Did this mean the detected cells were CD45PE negative or were genital tissue-resident cells?

*We thank the Reviewer for requesting clarification on our use of the term “tissue-resident.” In this manuscript, we do not measure tissue retention properties by these cells, and it is unlikely that recently immigrated cells would have upregulated these markers within the timeframe of pseudopregnancy (PMID: 24162776). Thus, in response to the Reviewer’s question, we have revised our use of this term in Figure 1 to state that we are instead measuring “tissue-localized cells.” Furthermore, we have revised Figure 2 and the legend to clarify that tissue-localized leukocytes are CD45 PE-CF594 negative but CD45 FITC+ leukocytes (IV negative: IV_neg_).*

3. In Figure 2 B-D, it appeared that most cells and cytokines peaked on day 6 after pseudopregnancy induction. Their levels on day 4 were lower than those on day 10. This trend was inconsistent with the data reported in Figure 5 and contradicted their claim that the increased susceptibility to C. muridarum on day 10 correlated with the decreased levels of cytokines and inflammatory cells.

*We apologize for not clearly stating our rationale and not providing sufficient data to support our focus on the early effector response following infection (DOI 3). In response to the Reviewer’s concern, we have added new experimental data that measures the early effector response to infection at DOI 3 by normalizing cell baseline values at that respective day of pseudopregnancy from uninfected mice in the revised Figure 5 C-F. This approach shows that day 8 challenge resulted in an increase in immune effector populations compared to pseudopregnancy alone; importantly, NK cells were increased at both the luminal surface and the LFRT as a result of infection. We agree with the Reviewer that day 4 and other time points are also interesting, and we intend to explore infection risk and pro-inflammatory immune dynamics occurring at these stages of the cycle in future studies. However, as we did not detect a difference in protection at DOI 3 following challenge at day 4 of pseudopregnancy, we did not explore day 4 further in this manuscript.*

4. Figure 4, based on the C. muridarum burden-shedding time courses presented in panes B&C, productive infection only occurred when C. muridarum was inoculated to MPA-treated mice or the pseudopregnancy-induced mice on day 10 after the induction since these two curves peaked on day 7 after infection. However, all inoculation conditions resulted in long-lasting shedding courses of chlamydial burden without clearance by the end of the observation on day 30 after infection. Obviously, the monitoring is not thorough enough to support unbiased conclusions. The authors must either prolong the observation time or measure viable organisms, which may allow the authors to acquire more comprehensive data to support unbiased conclusions.

*We thank the Reviewer for noting the need to better investigate the infection course following day 10 challenge, particularly at later time points to assess differences in clearance. Therefore, in the revised Figure 4 we added measurements of infectious elemental bodies, rather than relying solely on bacterial DNA, on days 21 and 28 of infection in mice initially challenged on day 8 or day 10 of pseudopregnancy. Additionally, we performed pathologic scoring of the FRT at day 45 post-infection (New Supplemental Figure 5) to confirm that these infections did not lead to tissue damage associated with chronic and ascending infections.*

5. Why did the authors only compare the cytokine and cell levels between day 8 and day 10 after pseudopregnancy induction? The day 4 and day 6 mice were as resistant to C. muridarum infection as the day 8 mice!!! It is unscientific to pick and choose data to support one’s model.

*We apologize for not more clearly stating our rationale in choosing to focus our comparisons between day 8 and day 10 of pseudopregnancy. As shown in the revised Figure 4, the only significant difference compared to MPA-treated mice at DOI 3 were mice challenged at day 8 of pseudopregnancy. There was also a significant difference between day 8 and day 10 pseudopregnant mice at DOI 28, which is why we also focused on that comparison. We have revised the text to better clarify our rationale for focusing on day 8. In addition, to address the Reviewer’s concerns we have added new data that support our rationale in Fig.2, Fig 5, and sFig 4, which show an enrichment of NK cells at the barrier site of pathogen exposure specifically in day 8 pseudopregnant mice.*

6. Figure 6, anti-NK1.1 depletion on day 8 after pseudopregnancy induction made the mice develop a shedding course similar to that of the MPA-treated mice. The role of NK1.1+ cells in controlling chlamydial infection in the female genital tract or systemic chlamydial infection has been demonstrated repeatedly in the literature. For example, Tseng and Rank depleted NK cells to increase chlamydial burden in MPA-treated mice (doi: 10.1128/iai.66.12.5867-5875.1998) while Xu et al used NK1.1 depletion to increase chlamydial burden in Rag1 KO mice (DOI: https://doi.org/10.1128/iai.00152-20). Although NK1.1+ cells may contain many different subsets of cells, since the NK1.1 depletion is always accompanied by a decrease in IFNg, the enhanced chlamydial burden has been attributed to the decrease in IFNg.

*The Reviewer is requesting more insight into the mechanisms driving NK cell-mediated protection during endometrial remodeling and how this differs from MPA-treated mice. In the revised manuscript, we performed transcriptomic analysis of FRT NK cells in pseudopregnant mice compared to MPA-treated mice in the revised Figure 6 using RNA sequencing. This approach shows that while NK cell populations are indistinguishable in circulation (spleen) between these groups, NK cells in the FRT exhibit clear differences in their programming. Specifically, FRT NK cells from MPA-treated mice are more enriched for genes associated with a resting state, while FRT NK cells from day 8 of pseudopregnancy are enriched for activation-associated programming, including ifng.*

7. NK cells are considered circulatory cells. The genital tract accessibility to circulatory cells peaked on day 8 after pseudopregnancy induction (Fig. 2). Why did the mice become susceptible to C. muridarum infection on day 10? The true mechanisms may be more complex than NK cells, addressing which may allow the authors to produce real new data.

*The Reviewer is requesting greater insight into the day 10 challenge time point. To address the Reviewer’s concern, we have added day 10 cellular data to the revised Figure 2. These data show that despite a risk of menses contamination in the lower FRT, there is a significant reduction in NK cells at day 10 of pseudopregnancy. We agree with the Reviewer that there are likely additional mechanisms that contribute to infection risk at this time point, but the decrease in NK cells is also likely a contributing factor given their importance for protection. We intend to further investigate the potential mechanisms that contribute to increased susceptibility at day 10 in future studies.*

**Reviewer #2**:

This manuscript by Lawrence at al. addresses a major research gap in the study of sexually transmitted infections, which is how immune responses are altered by the tissue remodeling processes that occur during a menstrual cycle. In light of the high prevalence and morbidity caused by of chlamydial infections in females of reproductive age and clinical observations that suggest reproductive hormones alter susceptibility and perhaps disease severity in women with C. trachomatis (Ct) infection, there is a major lack of fundamental immunological information in this area.

Strengths of this work include i.) the use of well-established pseudopregnancy model in mice to allow mimicking of the menstrual cycle in a murine model that is an accepted surrogate for Ct infection and disease in humans; ii.) the measurement of parameters over the course of the menstrual cycle in pig-tailed macaques to determine whether observations from the pseudo-pregnant mouse model are similar in a nonhuman primate; and iii) direct testing of one of the main hypotheses resulting from these first two studies using NK cell-depleted mice. The major finding that NK cells and IFN-gamma levels are highest during periods of lesser susceptibility and that NK cells play a protective role is important and may also be helpful in vaccine development. There is an enormous amount of data in this work and the assays used are sophisticated and state-of-the-art. In general, the figures are nicely prepared and convincing. The discussion is interesting. Weaknesses are few and suggestions are listed below.

**Part II – Major Issues: Key Experiments Required for Acceptance**
Please use this section to detail the key new experiments or modifications of existing experiments that should be absolutely required to validate study conclusions.

**Reviewer #2**: 1. Figure 1 does a good job at illustrating important aspects of the pseudopregnancy model. However, for readers who are trained in bacterial pathogenesis and not familiar with the female reproductive cycle, it might help to:
a. Define better what is going on during the decidualization phase and how it mimics what happens during pregnancy.
b. Panel D – include some arrows that point to the different cell types mentioned in the text. Are any erythrocytes visible in the picture of the menstruation phase?

*a-We thank the Reviewer for providing this valuable insight, and in addition to strengthening our rationale for phase stratification in the revised Figure 1, we added a direct comparison to a menstruating species using progesterone periodograms and waveform construction in the revised Figure 3E-F. We believe this new analysis provides clarity regarding the biological processes that occur during the different phases of the cycle.*

*b. We have now added arrows and an inset window to the revised Figure 1D to visualize and emphasize the presence of red blood cells during menstruation.*

2. Also, with respect to Figure 1,
a. Please state what is being compared in panels B and C in terms of the statistical differences (most likely the comparison is to MPA-treated mice, but this is not stated clearly here as it is in the other figures).
b. Please define the abbreviation “ssoil” that is used in the figure, either in the figure legend or better, write it out fully in the figure.
c. Panel H – multiple data points are shown within each bar, but the figure legend says the data are from a representative animal. This reviewer may not understand that assay, but it is wondering if each symbol corresponds from data from each animal within a group. And why are panels (B,C) mentioned on line 515. What are they referring to?

*a&b. We have revised panels B, C to better indicate the statistical comparisons, and we have defined the “ssoil” abbreviation.*

*c. We apologize for the confusion. The data shown in Figure 1G are from a representative mouse to illustrate the gating strategy that is applied to the measurements in Figure 1H, in which each dot corresponds to measurements from a single animal. We have now clarified this in the legend. In response to the Reviewer’s comment on (B, C), we have moved the strategy for statistical comparison to its appropriate location in the figure legend.*

3. Statistics: A more detailed description of the statistics used should be included in the Methods section. As the manuscript stands, most all of the all figure legends state
“Models used to compare a difference of means were fit using multiple comparisons”. What statistical test was used? What software was used?

*We thank the Reviewer for bringing this omission to our attention and have revised the material and methods to include the statistical testing/software. The statistical tests used for different comparisons are now listed in the respective figure legends*.

4. The use of the term(s) “circulating” or “in circulation” is not as accurate as saying plasma level (at least occasionally), in part so the reader knows what was measured. Example, lines 1222 … “progesterone levels in circulation had reached …” would be better stated as “plasma progesterone levels had reached…”

*We have corrected this sentence and checked for any other inappropriate use of this term.*

5. The Figure legends need figure titles instead of starting with each panel. For example, for Figure 2 – the figure title should say that the data are from mouse tissue and what the experiment(s) was in general (i.e. looking atr cell distribution with respect to anatomic site and cell types and cytokine levels with respect to day of sampling). For Figure 3, a figure title should say that the data are from the examination of naturally menstruating macaques… etc.

*We thank the Reviewer for providing this insight and have now added titles to all figures.*

6. Figure 4: Are these data from two separate experiments or one experiment with the same MPA control data being plotted in both panel B and panel C on different scales? If the results are from one experiment, all the data should be shown in one figure.

*We apologize for any confusion regarding the manner in which we initially displayed the data and have updated Figure 4B to combine all data into one graph.*

**Part III – Minor Issues: Editorial and Data Presentation Modifications**
Please use this section for editorial suggestions as well as relatively minor modifications of existing data that would enhance clarity.

**Reviewer #2**: - Why is progesterone and estrogen always capitalized within a sentence? This is distracting.

*We apologize for this error and have made sure progesterone is not capitalized within a sentence.*

- Line 37: “murine model of menstruation to investigate how endometrial shedding and repair alter the…” - alter should be alters

*We have corrected this typo.*

- Lines 75-78 “A major challenge in studying how menstruation impacts immune defenses at barrier sites is a lack of model systems that menstruate [15], especially common laboratory animal models that are supported by immunologic and genetic approaches that could facilitate mechanistic investigations into the dynamics of FRT tissue-localized immune cell populations [16-22]”. This is a beautiful concept but the sentence is long and complicated (could be two sentences) and the “supported by” phrase isn’t clear. Perhaps “especially common laboratory animal models with which immunological an genetic approaches can be used to facilitate….”

*We thank the reviewer for noting this and have corrected the sentence.*

-Line 92 C. muridarum (C. muridarum), a murine strain of chlamydia which models lower FRT infection by C. trachomatis [26, 27]”. The C. muridarum/mouse infection models upper FRT infection also, and very well compared to Ct in mice, including damage to the oviducts.

*We have corrected this sentence and added supporting citation.*

- Line 97 “…we found that while challenges administered under conditions of uterine repair resulted in robust infections, whereas challenges administered during…” – delete either “while” or “whereas”

*We have corrected this typo.*
